# Markers of nitric oxide are associated with sepsis severity: an observational study

**DOI:** 10.1186/s13054-017-1782-2

**Published:** 2017-07-15

**Authors:** Martin Sebastian Winkler, Stefan Kluge, Maximilian Holzmann, Eileen Moritz, Linda Robbe, Antonia Bauer, Corinne Zahrte, Marion Priefler, Edzard Schwedhelm, Rainer H. Böger, Alwin E. Goetz, Axel Nierhaus, Christian Zoellner

**Affiliations:** 1 Department of Anesthesiology, University Medical Center Hamburg-Eppendorf, Martinistr. 52, 20246 Hamburg, Germany; 2 Department of Intensive Care Medicine, University Medical Center Hamburg-Eppendorf, Martinistr. 52, 20246 Hamburg, Germany; 3 Institute of Clinical Pharmacology and Toxicology, University Medical Center Hamburg-Eppendorf, Martinistr. 52, 20246 Hamburg, Germany; 4 Center for Anesthesiology and Intensive Care Medicine, University Medical Center Hamburg-Eppendorf, Martinistr. 52, 20246 Hamburg, Germany

**Keywords:** L-arginine, Homoarginine, Asymmetric dimethylarginine, Dimethylarginase-dimethylalaminohydrolase-2, Sepsis

## Abstract

**Background:**

Nitric oxide (NO) regulates processes involved in sepsis progression, including vascular function and pathogen defense. Direct NO measurement in patients is unfeasible because of its short half-life. Surrogate markers for NO bioavailability are substrates of NO generating synthase (NOS): L-arginine (lArg) and homoarginine (hArg) together with the inhibitory competitive substrate asymmetric dimethylarginine (ADMA). In immune cells ADMA is cleaved by dimethylarginine-dimethylaminohydrolase-2 (DDAH2). The aim of this study was to investigate whether concentrations of surrogate markers for NO bioavailability are associated with sepsis severity.

**Method:**

This single-center, prospective study involved 25 controls and 100 patients with surgical trauma (n = 20), sepsis (n = 63), or septic shock (n = 17) according to the Sepsis-3 definition. Plasma lArg, hArg, and ADMA concentrations were measured by mass spectrometry and peripheral blood mononuclear cells (PBMCs) were analyzed for DDAH2 expression.

**Results:**

lArg concentrations did not differ between groups. Median (IQR) hArg concentrations were significantly lower in patient groups than controls, being 1.89 (1.30–2.29) μmol/L (*P* < 0.01), with the greatest difference in the septic shock group, being 0.74 (0.36–1.44) μmol/L. In contrast median ADMA concentrations were significantly higher in patient groups compared to controls, being 0.57 (0.46–0.65) μmol/L (*P* < 0.01), with the highest levels in the septic shock group, being 0.89 (0.56–1.39) μmol/L. The ratio of hArg:ADMA was inversely correlated with disease severity as determined by the Sequential Organ Failure Assessment (SOFA) score. Receiver-operating characteristic analysis for the presence or absence of septic shock revealed equally high sensitivity and specificity for the hArg:ADMA ratio compared to the SOFA score. DDAH2 expression was lower in patients than controls and lowest in the subgroup of patients with increasing SOFA.

**Conclusions:**

In patients with sepsis, plasma hArg concentrations are decreased and ADMA concentrations are increased. Both metabolites affect NO metabolism and our findings suggest reduced NO bioavailability in sepsis. In addition, reduced expression of DDAH2 in immune cells was observed and may not only contribute to blunted NO signaling but also to subsequent impaired pathogen defense.

## Background

Sepsis affects millions of people worldwide and its incidence is increasing [1]. A dysregulated immune response to an infection is the main feature of sepsis pathogenesis, which, together with hemodynamic and microcirculatory changes, may lead to insufficient tissue oxygenation, organ dysfunction, and septic shock [2, 3]. Nitric oxide (NO) is an important regulator of physiological processes in the immune system and circulation [4, 5]. NO is generated by a family of nitric-oxide synthases (NOS) within cells [6]. There are three tissue-specific isoforms termed endothelial (eNOS), neural (nNOS). and inducible NOS (iNOS), which are expressed in immune tissue. All NOS catalyze the production of NO from the substrates L-arginine (lArg) and homoarginine (hArg) [7]. NO levels are reduced when NOS is inhibited by asymmetric dimethylarginine (ADMA) in a competitive manner. ADMA levels again are controlled by dimethylarginase-dimethylalaminohydrolase-1 and 2 (DDAH1 and 2) activity, which inactivates ADMA by cleavage. DDAH2 is predominantly expressed in immune cells [8]. NO is a labile compound with a short half-life in blood making its direct detection and measurement unfeasible. Therefore, concentrations of substrates and endogenous inhibitors of NOS are considered surrogate markers for NO bioavailability [6].

NO levels in sepsis are critical and of particular interest for two reasons. First, NO regulates vascular function. Endothelial-derived NO dilates blood vessels by relaxing vascular smooth muscle (VSM). NO activates guanylate cyclase, which induces VSM relaxation by increasing intracellular 3,5-cyclic guanosine monophosphate (cGMP) concentration. In sepsis, NO can be thought of as a Janus-faced signaling molecule. On the one hand, excessive production of NO leads to severe hypotension and may cause signs of shock. On the other hand, NO is essential in maintaining microvascular function by regulating the supply and distribution of oxygen and nutrients throughout all tissues and organs [9]. In this context, NO maintains microvascular homeostasis by dilating and regulating vascular tone, red blood cell deformability, and leukocyte and platelet adhesion to endothelial cells [9]. Second, NO is essential in the immunological response to pathogens and has been extensively studied in macrophages [4]. NO is a free radical and has immediate antimicrobial effects including disruption of bacterial target structures and inhibition of bacterial metabolism (e.g. inactivation of the Krebs cycle or of virulence factors such as *Clostridium difficile* toxins A and B) [10–12]. Antimicrobial effects of NO play an important role in the response to infections. In mice infected with *Leishmania major,* NO was shown to suppress the metabolic activity of *L. major* without directly killing the pathogen but facilitated resolution of the disease by the immune response [13]. Recently, DDAH2, the ADMA-degrading enzyme in immune cells, has been studied in knockout mice. Global knockout of DDAH2 was associated with 80% lethality compared to wild-type animals when polymicrobial sepsis was induced, underlining the role of NO bioavailability in sepsis [14].

Considering the known effects of NO on vascular function and the immune system, we sought to investigate whether plasma concentrations of NOS substrates (lArg and hArg) and of the NOS inhibitor ADMA, and whether DDAH2 expression in immune cells are altered in patients with sepsis and whether the magnitude of any such alterations is related to disease severity.

## Methods

### Study population

From March to December 2014, 100 patients (>18 years old) who were admitted to the intensive care units (ICU) of the University Medical Center Hamburg-Eppendorf (Hamburg, Germany) with sepsis or after surgery were enrolled after informed consent had been obtained from patients or their legal representatives. The study cohort was previously described in detail [15] and analysis of metabolites from plasma samples and gene expression related to the Arg/NO-pathway were pre-specified in the study protocol approved by the local Research Ethics Committee (Hamburg Chamber of Physicians: reference PV4550).

Inclusion criteria were a diagnosed infection or a clinical syndrome pathognomonic for an infection. The former sepsis criteria published by the American College of Chest Physicians/Society of Critical Care Medicine were used in the original recruitment [16]. However, following publication of the consensus guidelines Sepsis-3 (which were not available during the study), patients were re-categorized to follow the more recent guidelines [17]. We generated Sequential Organ Failure Assessment (SOFA) scores for each patient and defined three groups: patients admitted to the ICU post-surgery were categorized as “surgical trauma”, patients admitted to the ICU with suspected or diagnosed infections were categorized as “sepsis”, and patients with hypotension requiring vasopressor therapy to maintain mean BP 65 mmHg or greater and with a plasma lactate concentration >2 mmol/L in spite of adequate fluid resuscitation were categorized as “septic shock” [3]. The control cohort consisted of 25 age-matched healthy volunteers.

### Clinical evaluations and assays

SOFA scores were calculated on admission and on day 3, if patients stayed at least 3 days in the ICU [18]. Within the first 24 h after inclusion, plasma samples were taken to measure lArg, hArg, and ADMA. We also collected peripheral blood mononuclear cells (PBMC) from patients and controls to analyze expression of DDAH2. Blood samples from controls and patients were processed identically. Plasma lArg, hArg, and ADMA concentrations were determined by liquid chromatography (LC)-tandem mass spectrometry (MS) analysis as described previously [18, 19]. Briefly, 25-μL aliquots of plasma were spiked with stable isotope-labeled hArg, lArg, and ADMA, which served as internal standards. Proteins were precipitated with 100 μL of methanol, filtered through a 0.22-μm hydrophilic membrane (Multiscreen HTS™, Millipore, Molsheim, France), derivatized with butanolic 1 N HCl, and analyzed by LC-tandem MS (Varian 1200 MS, Agilent Technologies, Santa Clara, CA, USA). Quantification was performed by calculation of peak area ratios and calibration with known concentrations of analytes in dialyzed EDTA plasma. Limits of quantification were 0.1 μmol/L for hArg, 0.25 μmol/L for lArg, and 0.005 μmol/L for ADMA. For all arginine metabolites, coefficients of variation were ≤7.5% [18, 19].

Collection of PBMC was performed using BD Vacutainer® CPT cell preparation tubes with sodium heparin (BD Bioscience, San Jose, CA, USA). PBMC were separated from other cell compartments according to the manufacturer’s protocol and stored at -80 °C in lysis buffer solution (RLT (Qiagen, Hilden, Germany) and 1% (v/v) beta-mercaptoethanol). Total RNA was prepared from PBMC using RNeasy Fibrous Tissue Mini Kit (Qiagen, Hilden, Germany). For real-time polymerase chain reaction (RT-PCR) analysis, single-strand complementary DNA (cDNA) was synthesized from 1 μg of total RNA sample isolated through TRIzol reagent (Invitrogen, Carlsbad, CA, USA) with a high capacity cDNA archive kit (Applied Biosystems, Foster City, CA, USA) as described previously [20]. Fifty nanograms of cDNA were amplified by RT-PCR using an ABI PRISM 7700 System and TaqMan reagents (Applied Biosystems) and normalized to glyceraldehyde 3-phosphate dehydrogenase (GAPDH) RNA as an endogenous control (TaqMan assay IDs: GAPDH, hs99999905_m1; DDAH2, hs00967863_g1).

### Statistical analysis

The primary variables were lArg, hArg, and ADMA concentrations in plasma in μmol/L and expression of DDAH2 in PBMC. DDAH2 values were first normalized to the internal control GAPDH and to expression levels in the control group using the delta-delta cycle threshold (Ct) method [21]. Variables were tested for normality of the distribution using the Shapiro-Wilk test. As all primary variables were not normally distributed, we report median and interquartile ranges (IQR). Differences between groups were tested for significance using either the non-parametric Mann-Whitney U test for two groups or Kruskal-Wallis analysis of variance (ANOVA) for more than two groups and trend analysis. Spearman’s rank correlation was used to analyze association between variables and clinical severity. Receiver-operating characteristic (ROC) curves were generated and areas under the curve (AUC) calculated. A *P* value <0.05 was considered to be significant. Statistical analyses were performed using SPSS® (vers. 21 IBM, Armonk, NY, USA) and Graph Pad Prism (version 7.0a GraphPad Software, La Jolla, CA, USA).

## Results

### Plasma homoarginine (hArg) levels were lower but ADMA levels were higher in sepsis

Briefly, the three study groups did not differ in age or sex distribution (Table 1). The surgical trauma group comprised 20 patients with postoperative inflammation after surgical trauma; these patients had undergone abdominal or thoracic surgery (n = 14) or other types of surgery (n = 6). The sepsis group comprised 63 patients, and the septic shock group comprised 17 patients (Table 1). Consistent with this classification, the ICU stay was longest for patients with septic shock, followed by patients with sepsis, and those with surgical trauma. The SOFA scores were highest in patients with septic shock followed by patients with sepsis and surgical trauma (Table 1).

**Table 1:** Baseline characteristics

	Controls	All patients	Surgical trauma	Sepsis	Septic shock
Number	25	100	20	63	17
Age, years^a^	49 (36–58)	60 (51–70)	61 (51–68)	60 (49–70)	60 (54–72)
Male, *n* (%)	15 (60)	58 (58)	11 (55)	37 (62)	10 (58)
SOFA score^a^	N/A	6 (3–8)	4 (2–7)	5 (3–7)	11 (8–13)
Length of ICU stay, days^a^	N/A	7 (2–11)	2 (1–5)	7 (3–10)	13 (8–31)

*SOFA* sepsis-related organ failure assessment score, *ICU* intensive care unit, *N/A* not applicable

^a^Data are presented as median (IQR)

There was no difference between the patient groups in median plasma lArg concentrations (Table 2). In contrast, the median plasma hArg concentration was lower in patients than in controls, being 1.89 μmol/L. Differences in median hArg levels showed a trend toward the greatest decreases in patients with more severe stages of sepsis. The lowest median plasma hArg concentration occurred in patients with septic shock, being 0.79 μmol/L and this was approximately 60% lower than in controls (*P* < 0.001; Table 2). Concentrations of the NOS inhibitor ADMA increased with clinical severity and were increased by 40% in sepsis with a median concentration of 0.80 μmol/L, and by 54% in septic shock with a median concentration of 0.89 μmol/L in patients compared with controls (*P* < 0.001; Table 2).

**Table 2:** Plasma arginine derivatives

	Controls	Surgical trauma	Sepsis	Septic shock	*P* value for trend^a^
L-arginine (μmol/L)	35.0 (21.6–52.9)	19.8 (13.0–48.5)	29.4 (14.8–42.5)	24.4 (6.2–49.3)	ns
Homoarginine (μmol/L)	1.89 (1.30–2.29)	1.06 (0.67–1.67)	0.92 (0.59–1.36)	0.79 (0.36–1.44)	<0.001
ADMA (μmol/L)	0.57 (0.46–0.65)	0.53 (0.44–0.65)	0.80 (0.56–0.93)	0.89 (0.56–1.39)	<0.001

Data are presented as median (IQR, interquartile range). *ADMA* asymmetric dimethylarginine, *ns* not significant

^a^Non-parametric Kruskal-Wallis test for trend analysis in controls vs. patient groups

### The hArg:ADMA ratio was correlated with organ failure

**Fig. 1:**
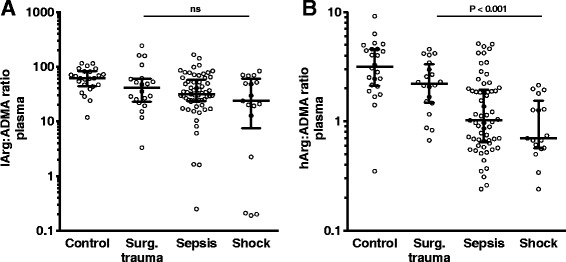
Ratios of L-arginine (*lArg*) (**a**) and homoarginine (*hArg*) (**b**) to asymmetric dimethylarginine (*ADMA*) in plasma samples from non-septic controls and in patients. Patients groups were compared using the non-parametric Kruskal-Wallis test for trend analysis between patients with surgical trauma (*Surg*), sepsis and septic shock (25 controls, 20 patients with surgical trauma, 63 patients with sepsis, and 17 patients with shock). Data are presented as median with interquartile range. *Circles* represent ratios of individual subjects. *ns* non-significant

**Fig. 2:**
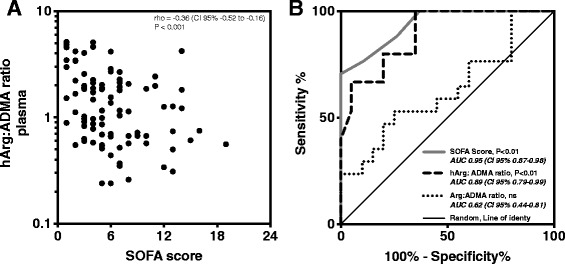
Association between the homoarginine (*hArg*) and asymmetric dimethylarginine (*ADMA*) ratio and the Sequential Organ Failure Assessment (*SOFA*) score, and as a predictive marker to differentiate septic shock from surgical trauma. **a** The plasma hArg:ADMA ratio was associated with the SOFA score. Spearman’s correlation analysis with the SOFA score as the dependent variable and plasma hArg:ADMA ratio are presented. Spearman’s rho and (95% confidence interval, *CI 95%*) are presented. **b** Receiver operating characteristic (ROC) curves for the identification of septic shock. Patients with septic shock were compared with patients with surgical trauma. ROC curves are shown for the SOFA score, plasma hArg:ADMA and L-arginine (*lArg:ADMA*) ratio. Areas under the curve (*AUC*) (95% CI) are presented with *P* values for significance. *ns* non-significant

**Fig. 3:**
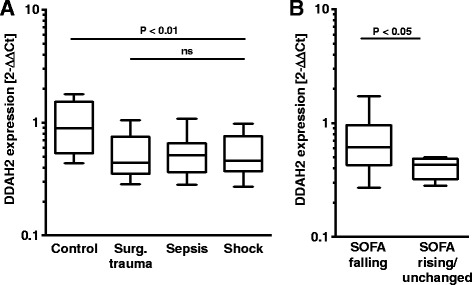
Dimethylarginine-dimethylaminohydrolase 2 (DDAH2) expression in peripheral blood mononuclear cells (PBMC). **a** DDAH2 expression levels in PBMC. DDAH2 expression was significantly lower in patients than controls (*P* < 0.01) but there was no difference between patient groups (non-significant (*ns*)). Total mRNA was prepared from PBMC and DDAH2 expression levels were assessed by quantitative PCR (qPCR) in controls (n = 22) and in patients (n = 45). **b** Patients with declining Sequential Organ Failure Assessment (*SOFA*) scores (delta-SOFA) from day 1 to day 3 (n = 22) were compared with patients with rising or unchanged delta-SOFA (n = 5). DDAH2 expression was lower in patients with rising SOFA than in patients with falling or unchanged SOFA (*P* < 0.05). The relative expression values were calculated using glyceraldehyde 3-phosphate dehydrogenase (GAPDH) as an internal standard and are presented by the delta-delta cycle threshold (*Ct*) method using the mean value of controls for standardization. The *horizontal line* in the *box* is the median value; the *box* extends to the interquartile range (25–75th centile) and the *whiskers* to the minimum and maximum values. The group differences were calculated using the non-parametric Kruskal-Wallis test for trend analysis for more than two gropus and non-parametric Mann-Whitney U test for two groups.

Since lArg and hArg are NOS substrates and ADMA is the endogenous NOS inhibitor, we calculated the plasma lArg:ADMA and plasma hArg:ADMA ratios. Only the plasma hArg:ADMA ratio correlated with clinical severity and differentiated between clinical stages (Fig. 1a and b). The median plasma hArg:ADMA ratio was significantly lower in patients with shock than in patients with surgical trauma or sepsis (Fig. 1b; *P* < 0.001). To investigate whether plasma hArg:ADMA ratio correlated with the severity of sepsis as measured by the SOFA score we performed Spearman’s rank correlation analysis. Plasma hArg:ADMA ratios were inversely correlated with SOFA scores with a rho of -0.36 (Fig. 2a; *P* < 0.001). ROC analysis was performed to further demonstrate the potential of plasma lArg:ADMA and hArg:ADMA ratios to differentiate septic shock from surgical trauma. The plasma hArg:ADMA ratio emerged as an indicator of septic shock with almost identical AUCs to those for the SOFA score (0.95 vs. 0.89, respectively; Fig. 2b), whereas the lArg:ADMA ratio with an AUC of 0.69 was not significant (Fig. 3b).

### DDAH2 expression was lower in patients and correlated with disease severity

As ADMA is cleaved by DDAH2 in immune cells, we were interested in whether DDAH2 expression is altered in PBMC. We observed significantly lower levels of DDAH2 expression in patient groups than in controls (Fig. 3a). Dividing the cohort into patients with either falling or rising/unchanged SOFA between day 1 and day 3, we observed lower expression of DDAH2 in patients with rising/unchanged SOFA scores (Fig. 3b).

## Discussion

We found that plasma hArg levels were significantly reduced whereas lArg levels remained unchanged in patients with sepsis. Plasma ADMA levels were increased in patients with sepsis; and both hArg and ADMA were associated with sepsis severity. Taken together the plasma hArg:ADMA ratio was increasing, and was closely associated with septic shock.

Sepsis is a systemic inflammatory response to an infection [2, 3]. The two most important factors affecting sepsis outcome are circulatory failure and immune suppression. NO is a pivotal signaling molecule with regulatory functions in both the circulation and the immune response. In this context, we hypothesized that decreased NO bioavailability in sepsis may contribute to disease progression. Physiologically NO regulates blood pressure. In vascular disease associated with hypertension such as atherosclerosis, coronary heart disease, or chronic renal failure *low* substrate levels but *high* inhibitor levels of NOS are markers of disease severity and mortality [22]. This is supported by experimental observations in animals and humans; supplementation of lArg in rabbits can restore vascular relaxation and endothelial function, whereas in humans infusion of ADMA results in increased systemic vascular resistance (SVR) and mean arterial blood pressure (MAP) [23–26]. In contrast, the hallmark of sepsis and septic shock is reduced oxygen delivery depending on alteration in cardiac output (CO), the product of SVR and MAP [27]. This leads to the hypothesis that excessive NO production may contribute to reduced SVR and MAP [28]. Therefore it was appealing to test NOS inhibitors in sepsis to restore oxygen delivery. A randomized clinical trial compared intravenous administration of the non-selective NOS inhibitor L-N^G^-methyl-L-arginine hydrochloride (546C88) with placebo. Indeed, 546C88 directly increased SVR but cardiac output and oxygen delivery were subsequently blunted [29]. Nonetheless, in the long term, administration of 546C88 was associated with higher mortality [30]. This can be explained by the fact that systemic inhibition of NOS in all tissues might be responsible for reducing signs of hypotension but may have an impact on capillary exchange or the immune response [6]. An alternative therapeutic concept might be the inhibition of the downstream signaling of NO, e.g. by methylene blue [31–33].

We found lower levels of hArg in patients with sepsis than in controls but unchanged levels of lArg, which is remarkable. K_m_ values of hArg are much higher than those for lArg [34]. One may argue that hArg is less relevant than lArg and most studies in sepsis have focused on lArg. However, the usefulness of lArg as a sepsis marker is questionable as results of various clinical studies are inconsistent. In a group of 44 patients with cardiogenic or septic shock, lArg levels were shown to remain unchanged and in a randomized trial including 267 sepsis patients no changes in lArg levels were reported [35, 36]. In addition, a longitudinal study of 60 patients with septic shock observed no changes in lArg levels within the first 24 h but increased lArg levels at day 4 [37]. This suggests that the timing of measuring lArg in blood might be critical and usefulness as an early sepsis marker is questionable. To investigate hArg levels was one purpose of our study as data for hArg levels in sepsis are limited. Recent studies indicate that hArg is also involved in vascular homeostasis. According to a population-based cohort of 746 elderly participants, hArg and lArg are independently and antagonistically associated with blood pressure [38]. Of note, in interventional studies in mice, hArg supplementation has been shown to improve neurological outcome and cardiac function [39, 40]. Interestingly in kinetic experiments, when healthy humans were injected with lipopolysaccharide (LPS), NO-dependent vasodilatation increased but no changes in the concentrations of lArg in the blood were observed [41]. Therefore, systemic NO levels in blood during sepsis may not be lArg but hArg dependent. Unfortunately, the study cited did not report hArg levels. However, if hArg has a higher K_m_ value than lArg, we do not know if the catalytic efficiency of NOS is altered or if different NOS isoforms are preferentially metabolizing hArg during systemic inflammation and sepsis, which would explain why low hArg but not lArg levels correlated with disease severity in our study.

Despite NO effects in the circulation, reduced levels of NO in sepsis might be harmful for another reason. Cecal ligation reduces survival in NOS knockout mice compared to wild-type animals [31]; and bone marrow transplantation of wild-type to NOS knockout mice increases release of cytokines such as TNF-α and was found to improve survival in a model of idiopathic pneumonia [32]. Together with the discovery that mouse macrophages produce large amounts of nitrite (NO2-) and nitrate (NO3-) upon LPS stimulation, it has been suggested that decreased NO levels may impair the innate immune response [33], which is characteristic of sepsis progression in humans. Human monocytes show diminished defense mechanisms. For instance, the reduced ability to release pro-inflammatory cytokines after endotoxin stimulation has been referred to as monocyte “anergy” [42]. In this context it has been shown that hArg can serve exclusively as a substrate for NOS in macrophages [43]. Interestingly, the pharmacological transformation of hArg to the NOS inhibitor NH2-homoarginine results in significantly decreased NO production by macrophages [44]. One may speculate that decreased hArg levels may indicate monocyte “anergy” and this may explain why the hArg:ADMA ratio may be better than the lArg:ADMA ratio for diagnosing sepsis severity.

In addition to substrate availability, NOS activity is also regulated by the presence of NOS inhibitors. ADMA is an endogenous NOS inhibitor and increased levels are associated with decreased NO bioavailability. Other authors have hypothesized that increasing ADMA levels may contribute to poor sepsis outcome. For instance, in a cohort of patients with sepsis caused by the malaria parasite *Plasmodium falciparum,* ADMA was increased and associated with mortality [45]. Recently, findings from another sepsis study have suggested that ADMA may provide a non-invasive measurement of microvascular function. ADMA levels were not only increased and associated with the SOFA score, but pathologic values of the reactive-hyperemia index to estimate microvascular function were associated with a decreasing lArg to ADMA ratio [46], and in the aforementioned randomized controlled trial increased ADMA levels were independently associated with 90-day mortality [36].

However, it is unknown exactly how ADMA levels are regulated in sepsis. ADMA is metabolized to citrulline and dimethylamine by DDAH and enters cells through cationic amino-acid transporters (CAT). Two isoforms of DDAH are expressed in humans. DDAH1 predominates and is extensively expressed in the liver, lungs, and kidneys [47]. DDAH2 is primarily expressed in endothelial and immune cells [8, 48]. Circulating ADMA levels are genetically determined by promoter polymorphism in a regulatory gene encoding DDAH2 polymorphism and have been investigated by other research groups. Interestingly, in a cohort of 236 patients with postoperative inflammation after elective cardiac surgery, the DDAH2 -449G allele was identified as a polymorphism associated with an increased requirement of vasopressor to maintain organ perfusion [49]. In another series of 47 patients with severe sepsis, high ADMA concentration was also associated with the DDAH2-449G polymorphism [50]. In contrast, in pediatric sepsis, the DDAH2-449G polymorphism was associated with low ADMA concentration but with an increased likelihood of “cold” shock [51]. However, children with sepsis have much more variable hemodynamic profiles often with increased incidence of low cardiac output and elevated vascular resistance, which makes pediatric sepsis different from adult sepsis [52]. Interestingly, we found an association between decreasing DDAH2 mRNA expression in PBMC and disease severity. This is intriguing as animal experiments in global DDAH2-knockout mice have shown unchanged systemic ADMA and NO concentrations compared to wild-type mice. However, knockout mice injured by cecal ligation had 120-h survival of only 12% compared to 53% in wild-type animals [14]. The authors attributed this phenotype to impaired macrophage function. Monocyte-specific deletion of DDAH2 results in a similar pattern of increased severity to that seen in globally DDAH2-deficient animals. DDAH2 knockout in macrophages was associated with a significantly higher bacterial load in plasma and the peritoneum. Lambden and colleagues have shown that NO production in activated mouse macrophages is DDAH2-dependent with reduced intracellular NO levels within the cell. Moreover, DDAH2 knockout also impaired motility and phagocytosis in these cells.

With data from our small cohort we cannot conclude that decreased DDAH2 expression is monocyte or macrophage specific, as PBMC contain not just blood monocytes, but also B cells, dendritic cells, and activated T cells, which is a limitation of our expression findings. Limitations of our study are that it was carried out at a single center and involved relatively small numbers of patients, and larger cohorts should eliminate controls and potential selection bias. However, we observed associations between disease severity and systemic inflammation and sepsis, with surrogate markers of NO metabolism indicating decreased NO bioavailability in sepsis. To explain why hArg is decreased and ADMA is increased in sepsis requires further experimental studies. Nevertheless, we believe that our observations warrant follow-up studies with larger patient groups to confirm the power of the hArg:ADMA ratio to predict septic shock and sepsis severity.

## Conclusion

Taken together, the close association between decreased hArg:ADMA ratio and sepsis severity in our cohort may hint at perturbation of endothelial function and pathogen defense. Impaired endothelial function and defense mechanisms due to decreased NO production in endothelial and immune cells may be a mechanism linking hArg, ADMA, and DDAH2 expression with organ dysfunction and impaired immune response in sepsis. The DDAH2-ADMA axis is a potential target and may be important in individual tailoring of therapy. Agents that compete with ADMA for NOS (such as hArg) or that potentiate DDAH2 activity should be further investigated in sepsis.

## Key messages

- Homoarginine is a substrate of NOS and together with endogenous NOS inhibitor ADMA its ratio is reduced in sepsis in proportion to sepsis severity
- Decreased DDAH2 expression in PBMC is associated with sepsis outcome

## Abbreviations

ADMA: Asymmetric dimethylarginine
ANOVA: Analysis of variance
AUC: Areas under the curve
cDNA: Single-strand complementary DNA
cGMP: 3,5-Cyclic guanosine monophosphate
CO: Cardiac output
DDAH: Dimethlyarginine-dimethylalaminohydrolase
GAPDH: Glyceraldehyde 3-phosphate dehydrogenase
hArg: Homoarginine
lArg: L-arginine
LC: Liquid chromatography
LPS: Lipopolysaccharide
MAP: Mean arterial pressure
MLCP: Myosin light chain phosphatase
MS: Mass spectrometry
NO: Nitric oxide
NOS: Nitric-oxide synthase
PBMC: Peripheral blood mononuclear cells
RNA: Ribonucleic acid
ROC: Receiver-operating characteristic curve
RT-PCR: Real-time polymerase chain reaction
SD: Standard deviation
SOFA: Sequential Organ Failure Assessment
TNF: Tumor necrosis factor
VSM: Vascular smooth muscle
